# The effects of transcranial direct current stimulation on EEG power and complexity of ischemic stroke

**DOI:** 10.3389/fnins.2025.1688032

**Published:** 2025-12-10

**Authors:** Wang Xi, Guoqiang Song, Zhizhao Ma, Liqiang Liu

**Affiliations:** Department of Neurosurgery, The Second Hospital of Hebei Medical University, Shijiazhuang, China

**Keywords:** transcranial direct current stimulation, ischemic stroke, neural oscillations, relative power, Lempel-Ziv complexity, sample entropy

## Abstract

Transcranial direct current stimulation (tDCS), has shown therapeutic potential for ischemic stroke, but the neural oscillatory mechanisms of its therapeutic effects remain incompletely elucidated. Based on the rat model and clinical stroke patients, this paper combines multiple indicators such as electroencephalography (EEG) relative power (RP), Lempel-Ziv complexity and sample entropy to investigate the mechanism by which tDCS modulates neural oscillations in ischemic stroke. The results indicated that tDCS substantially reduced the RP of the *δ* frequency band, enhanced the normal oscillation of the *α* frequency band, and improved the complexity index of the EEG signals. These findings suggest that tDCS could facilitate the recovery following ischemic stroke through the modulation of EEG rhythms.

## 1 Introduction

The Global Burden of Disease Study identifies stroke as the second leading cause of mortality and the third most prevalent cause of disability worldwide (Feigin et al., 2021). Among the different types of stroke, ischemic stroke constitutes approximately 85% of cases, resulting in approximately 6.4 million deaths each year (Xie et al., 2024). Most survivors not only face the challenge of physical disability, but also the difficulty of cognitive dysfunction, which imposes a significant burden on their families as well as society.

Conventional rehabilitation strategies for ischemic stroke are mainly based on drugs and cognitive interventions, which exhibit limited efficacy in the clinical treatment process (Winstein et al., 2016; Wolf et al., 2021). With the accelerated progress of non-invasive brain stimulation in recent years, unprecedented opportunities have emerged for the modulation of brain function. Transcranial direct current stimulation (tDCS) has seen growing application the field of neurorehabilitation by regulating the excitability of cerebral cortical neurons through the continuous application of weak currents to the scalp (Chase et al., 2020). Previous investigations have indicated that tDCS can promote improvements in motor, cognitive and language functions of stroke animals (Huang et al., 2025; Zhang et al., 2020) or patients (Bornheim et al., 2020; Bornheim et al., 2022; Fridriksson et al., 2018). However, the neural oscillatory mechanisms underlying the action of tDCS in ischemic stroke have not been elucidated.

Neural oscillations reflect the synchronized activity of neuronal clusters in the brain, and the oscillations in different frequency bands show different functional significance (Buzsáki and Draguhn, 2004). Studies have shown that excessive synchronization of low-frequency oscillations (*δ*, *θ*) may be related to brain injury and motor recovery, and it could qualify as a biomarker in stroke recovery and rehabilitation (Cassidy et al., 2020; Gyulai et al., 2021; Rabiller et al., 2015). Meanwhile, *α* and *β* oscillations are related to motor coordination and can also serve as features for assessing post-stroke movement disorders (Adam et al., 2015; Avanzini et al., 2012). However, power only describes energy distribution and cannot measure the predictability and complexity of time series. Nonlinear dynamic indicators—Lempel-Ziv complexity (LZC) and sample entropy (SampEn)—precisely fill this gap: they are closely related to neural plasticity and recovery (Cheng et al., 2019; Liu Y. et al., 2023) and can sensitively capture changes in neural complexity in the early stages of stroke (Zhu et al., 2024). Additionally, they can serve as electrophysiological predictors of functional recovery after stroke (Li et al., 2020). Exploring the changes in these indicators can provide novel insights into comprehending the intervention mechanisms of tDCS in ischemic stroke.

This study aims to systematically reveal the neural oscillation regulation mechanism of tDCS in ischemic stroke. It combined the rat middle cerebral artery occlusion (MCAO) model with cases of stroke patients and used electroencephalography (EEG) multi-parameter analysis to provide a theoretical basis for individualized rehabilitation treatment.

## 2 Materials and methods

### 2.1 Subjects

Animal group: Forty Sprague–Dawley rats (male, 8 weeks) procured from Beijing Vital River Laboratory Animal Technology Co., Ltd. were allocated equally into 4 groups: Control, MCAO, MCAO + tDCS, and MCAO + Sham. After the rats were anesthetized, the neck vessels were separated and exposed, and a nylon thread was then introduced via the bifurcation of the common carotid artery into the internal carotid artery, blocking the origin of the middle cerebral artery (MCA) and all its collateral blood supply, resulting in focal ischemia in the MCA area. This completed the preparation of the ischemic stroke animal model. A tungsten wire electrode (80 μm in diameter) was implanted in the motor cortex (anteroposterior: 1.5 mm, mediolateral: 2 mm, dorsoventral: −1 mm from bregma), and two screw electrodes were positioned in the cerebellar region to serve as the reference and ground electrodes. All electrodes were fixed on the skull with dental cement to facilitate the collection of EEG signals (Mumtaz et al., 2018; Yoo et al., 2021). Following the surgical procedure, the rats were returned to a controlled environment characterized by stable temperature (20–25 °C) and humidity (55 ± 15%) conditions, a 12-h light cycle, and a sufficient supply of food and water, all within a barrier enclosure.

Clinical group: Twelve patients with ischemic stroke (males, age range 58–71) were recruited from the Second Hospital of Hebei Medical University and randomly allocated into the Stroke group (*n* = 6, ages 59–70) and the Stroke + tDCS group (*n* = 6, ages 58–71). Additionally, six healthy volunteers were included as Control group (male, *n* = 6, ages 58–70). All participants signed informed consent.

### 2.2 tDCS protocol

Animal group: 24 h after MCAO surgery, tDCS was applied for 7 consecutive days, 20 min/day, once a day, at a current of 0.2 mA. During this period, the anodal and cathodal electrodes were positioned over the motor cortex and occipital region, respectively.

Clinical group: The anodal electrode of the tDCS (Sichuan Intelligent Electronics Industrial Co., Ltd., IS200) was positioned over the motor cortex, and the cathodal electrode was positioned over the frontal area of the healthy side. The current intensity was 1.5 mA, applied for 7 consecutive days, 20 min/day, once a day.

### 2.3 EEG recording

On the second day after the tDCS intervention, 30 min of continuous EEG signals were collected while the animals and participants were in an awake state.

Animal group: The multi-channel EEG signal acquisition system (Apollo, Bio-Signal Technologies, United States) was utilized to record EEG signals.

Clinical group: The wearable wireless EEG signal acquisition system (Neuracle, Bio-Signal Technology (Changzhou) Co., Ltd., China) was utilized to record EEG signals.

### 2.4 EEG data analysis

MATLAB (R2022b) software was used to analyze entire EEG signals across distinct frequency bands, including *δ* (1–4 Hz), *θ* (4–8 Hz), *α* (8–13 Hz), and *β* (13–20 Hz). The specific indicators are as follows:

(1) Relative power (RP)

RP is defined as the proportion of power within a specific frequency band to the total power across all frequency bands. It is used to quantify the energy distribution characteristics of different frequency components in EEG signals (Quinn et al., 2019). The specific calculation is presented in Equation 1:


(1) $$RP_{\text{band}}=\frac{P_{\text{band}}}{\sum P_{\text{total}}}\times 100\%$$

(2) Lempel-Ziv complexity (LZC)

LZC quantifies signal complexity through analyzing the frequency of novel patterns in time series, reflecting the randomness and predictability of EEG signals (Lempel and Ziv, 1976). A higher value indicates a more complex signal. The normalized complexity is presented in Equation 2:


(2) $$\mathrm{LZC}=\frac{c(n)}{n}\log_{m!}n$$

Where c(n) is the complexity, m represents the number of data points in each pattern, n denotes the length of the symbol sequence, m! signifies the number of possible patterns.

(3) Sample entropy (SampEn)

Sample entropy evaluates the likelihood of pattern recurrence in a time series (Richman and Moorman, 2000). A smaller value indicates a more regular signal. The specific calculation is as shown in Equations 3–5:


(3) $$\begin{array}{c} \text{SampEn}(m,r,N)=-\ln[\frac{A^{\left(m\right)}(r)}{B^{\left(m\right)}(r)}] \end{array}$$

(4) $$\begin{array}{c} B^{\left(m\right)}(r)=\frac{1}{N-m}\sum_{i=1}^{N-m}C_{i}^{m}(r) \end{array}$$

(5) $$\begin{array}{c} A^{\left(m\right)}(r)=\frac{1}{N-m}\sum_{i=1}^{N-m}C_{i}^{m}(r) \end{array}$$

In the aforementioned formula, $B^{\left(m\right)}(r)$ and $A^{\left(m\right)}(r)$ represent the total number of template matches for embedding dimensions m , and m+1 , respectively. The index i corresponds to data points, m denotes the embedding dimension, r signifies the tolerance or similarity radius, N denotes the length of the time series, and $C_{i}^{m}(r)$ denotes the probability that the vector distances are less than r .

### 2.5 Statistical analysis

Statistical analysis was performed using IBM SPSS Statistics 27 software, with data presented as mean ± standard error of mean (SEM). Following confirmation of normal distribution and homogeneity of variance assumptions, one-way ANOVA was employed to compare data across different groups of animals and patients. Statistical significance was defined as a *p*-value < 0.05 (*p < 0.05, **p < 0.01, ***p < 0.001). Eta-squared (η^2^) was used to calculate effect sizes (Fields et al., 2023).

## 3 Results

### 3.1 The regulatory effect of tDCS on the RP in ischemic stroke

**Figure 1:**
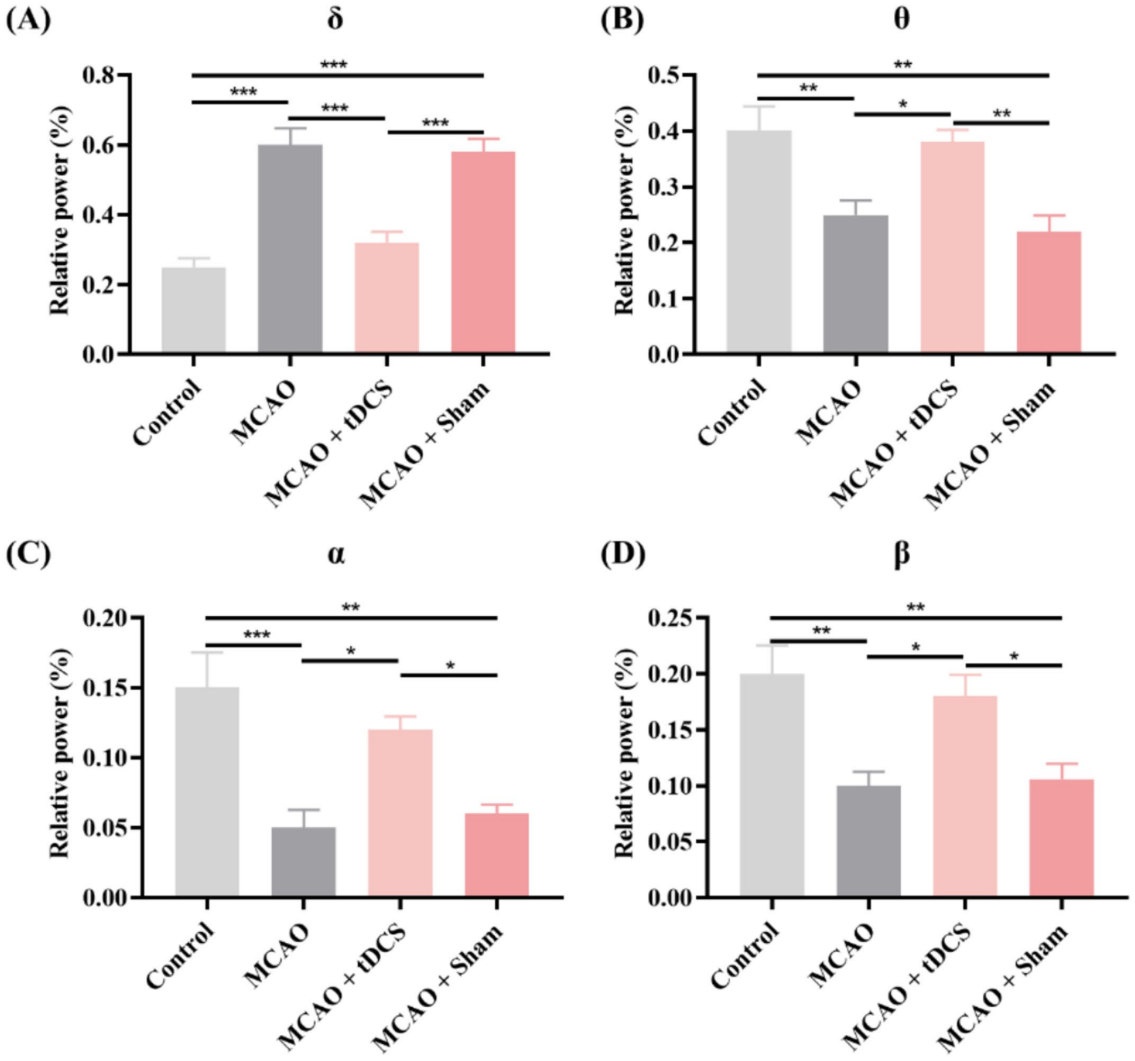
The RP of **(A)** *δ*, **(B)** *θ*, **(C)** *α*, and **(D)** β bands in the animal groups. (mean +SEM, One-way ANOVA, *p < 0.05, **p < 0.01, ***p < 0.001).

To evaluate the effect of tDCS on ischemic stroke, the RP was analyzed. The alterations in RP across distinct rat groups are presented in Figure 1. The results revealed a notable elevation in the RP of the *δ* frequency band within the MCAO group. And after the tDCS intervention, the RP of this frequency band was significantly reduced. The RP of the *θ*, *α*, and *β* bands decreased significantly after ischemic stroke. After tDCS was applied, the RP of these bands increased significantly and returned to nearly normal levels. The group of MCAO + Sham exhibited a similar state to that of the MCAO group (mean ± SEM, One-way ANOVA, *δ*: *p* < 0.001, η^2^ = 0.666; *θ*: *p* < 0.05, η^2^ = 0.413; *α*: *p* < 0.05, η^2^ = 0.452; *β*: *p* < 0.05, η^2^ = 0.392).

**Figure 2:**
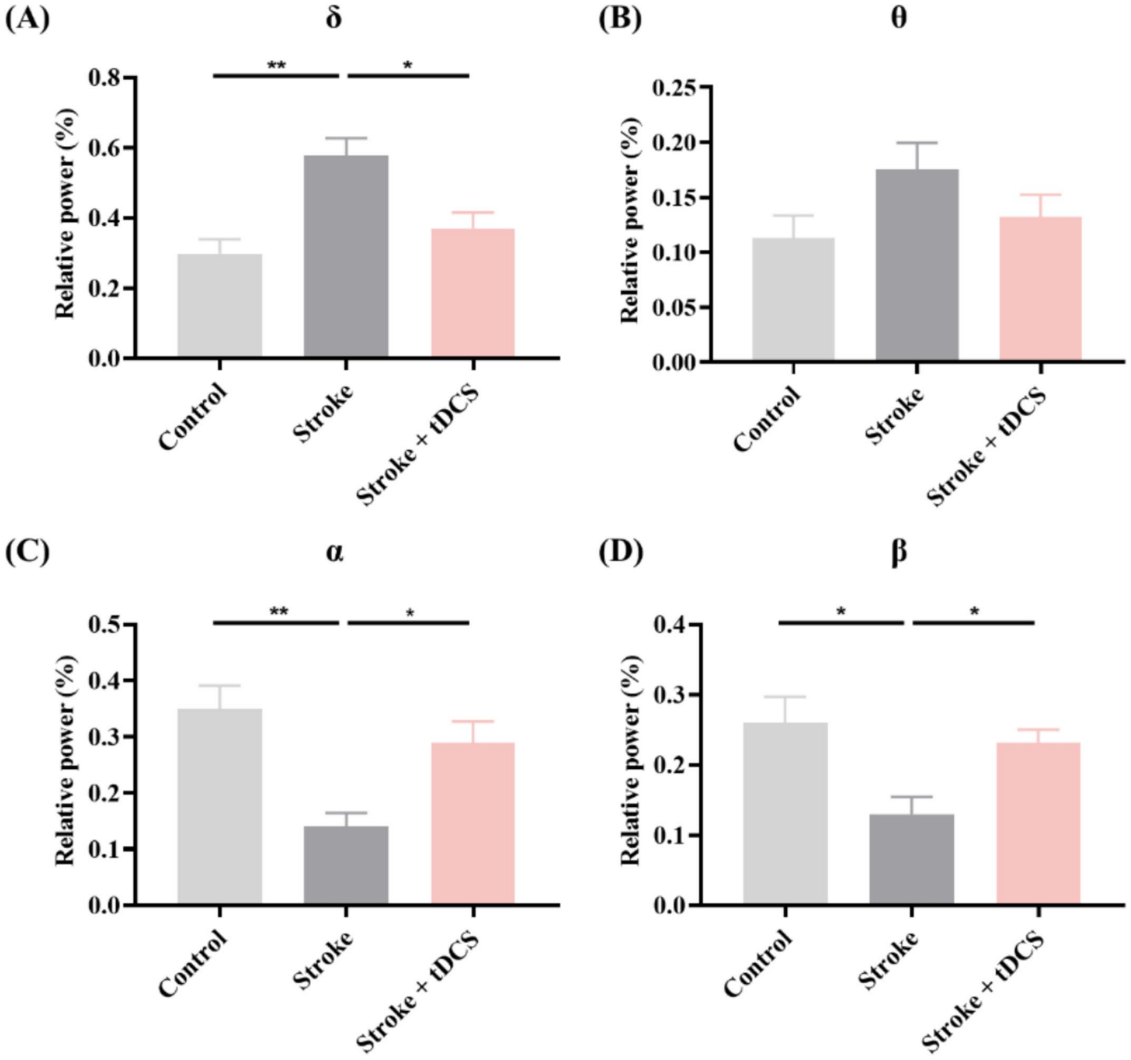
The RP of **(A)** δ, **(B)** θ, **(C)** α, and **(D)** β bands in the clinical groups (mean +SEM, One-way ANOVA, *p < 0.05, **p < 0.01).

Further, we applied tDCS to patients with ischemic stroke. As depicted in Figure 2, the Stroke group exhibited significantly higher RP in the δ band. After the tDCS intervention, the RP of this band was significantly reduced. While the RP of the θ band showed an upward trend in the Stroke group, the difference did not reach statistical significance. The RP of the α and β frequency bands decreased notably in the Stroke group and increased markedly following the application of tDCS (mean ± SEM, One-way ANOVA, *δ*: *p* < 0.05, η^2^ = 0.582; *α*: *p* < 0.05, η^2^ = 0.564; *β*: *p* < 0.05, η^2^ = 0.449).

These findings suggest that tDCS can effectively regulate neural oscillation activity in ischemic stroke.

### 3.2 The regulatory effect of tDCS on the LZC in ischemic stroke

**Figure 3:**
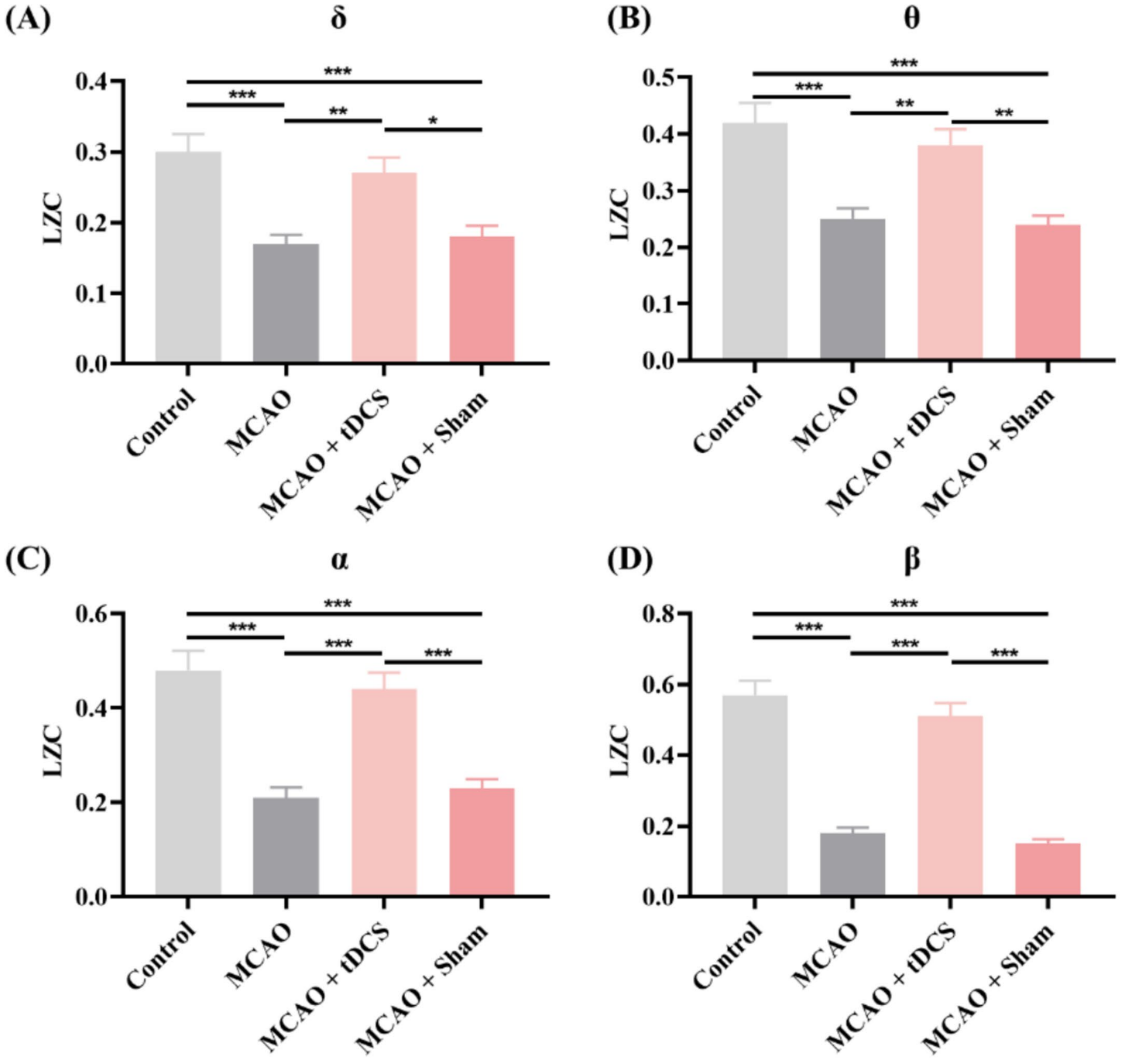
The LZC of the animal groups in the **(A)** δ, **(B)** θ, **(C)** α, and **(D)** β frequency bands. (mean +SEM, One-way ANOVA, *p < 0.05, **p < 0.01, ***p < 0.001).

Next, we analyzed the LZC. Figure 3 shows the changes in LZC in rat groups. The results revealed a notable reduction in the LZC across all frequency bands in the MCAO group. After the tDCS intervention, the LZC recovered significantly, whereas the group of MCAO + Sham exhibited a similar state to that of the MCAO group (mean ± SEM, One-way ANOVA, *δ*: *p* < 0.05, η^2^ = 0.476; *θ*: *p* < 0.01, η^2^ = 0.512; *α*: *p* < 0.001, η^2^ = 0.635; *β*: *p* < 0.001, η^2^ = 0.818).

**Figure 4:**
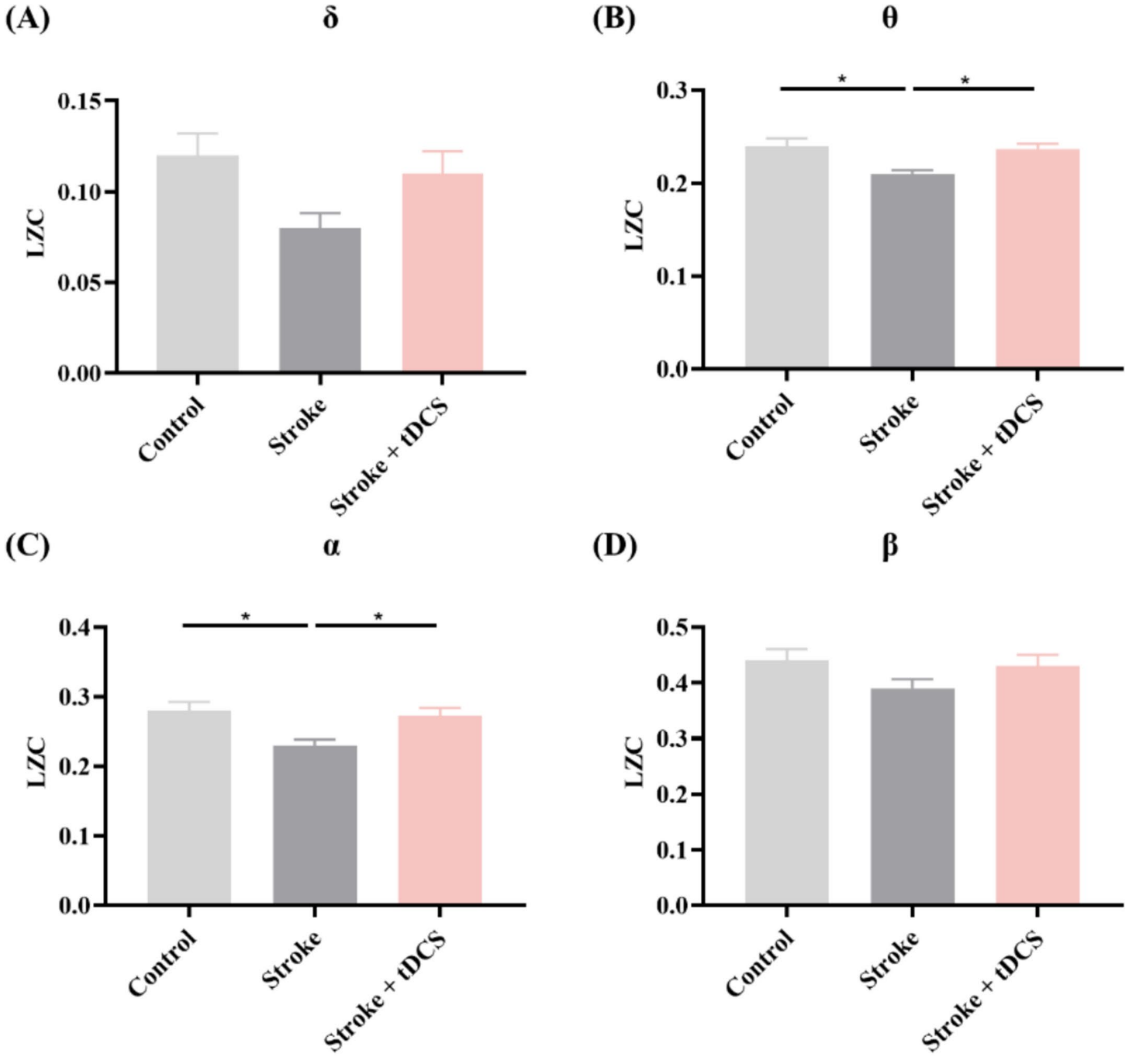
The LZC in the **(A)** δ, **(B)** θ, **(C)** α, and **(D)** β frequency bands of the clinical groups (mean +SEM, One-way ANOVA, *p < 0.05).

The LZC of the Stroke group also showed a downward trend. In particular, notable differences were identified in the *θ* and *α* frequency bands. After the tDCS intervention, both frequency bands exhibited significant recovery effects (mean ± SEM, One-way ANOVA, *θ*: *p* < 0.05, η^2^ = 0.482; *α*: *p* < 0.05, η^2^ = 0.440, Figure 4).

These results indicated that tDCS effectively improves the complexity of EEG signals in both animal models and clinical patients with ischemic stroke. This finding may be related to the alleviation of neural network damage in stroke.

### 3.3 The regulatory effect of tDCS on the SampEn in ischemic stroke

**Figure 5:**
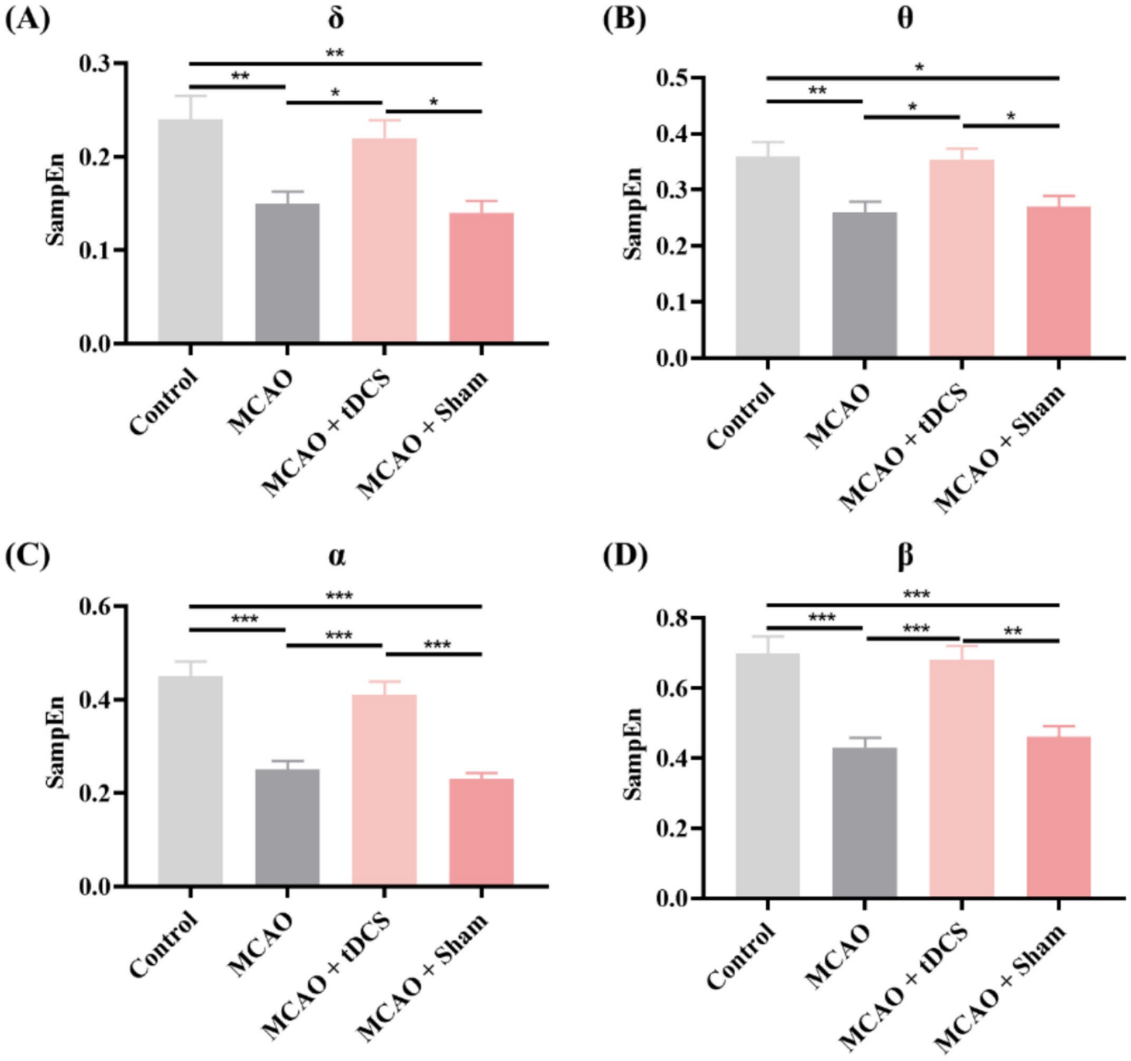
The SampEn in the **(A)** δ, **(B)** θ, **(C)** α, and **(D)** β frequency bands of the animal groups (mean +SEM, One-way ANOVA, *p < 0.05, **p < 0.01, ***p < 0.001).

Finally, we analyzed the SampEn. Figure 5 shows the changes in SampEn between different groups of rats. The findings were comparable to the LZC results. The SampEn of each frequency band in the MCAO group was significantly decreased. Following the tDCS intervention, the SampEn significantly recovered, while the group of MCAO + Sham exhibited no notable changes (mean ± SEM, One-way ANOVA, *δ*: *p* < 0.05, η^2^ = 0.386; *θ*: *p* < 0.05, η^2^ = 0.352; *α*: *p* < 0.001, η^2^ = 0.639; *β*: *p* < 0.01, η^2^ = 0.540).

**Figure 6:**
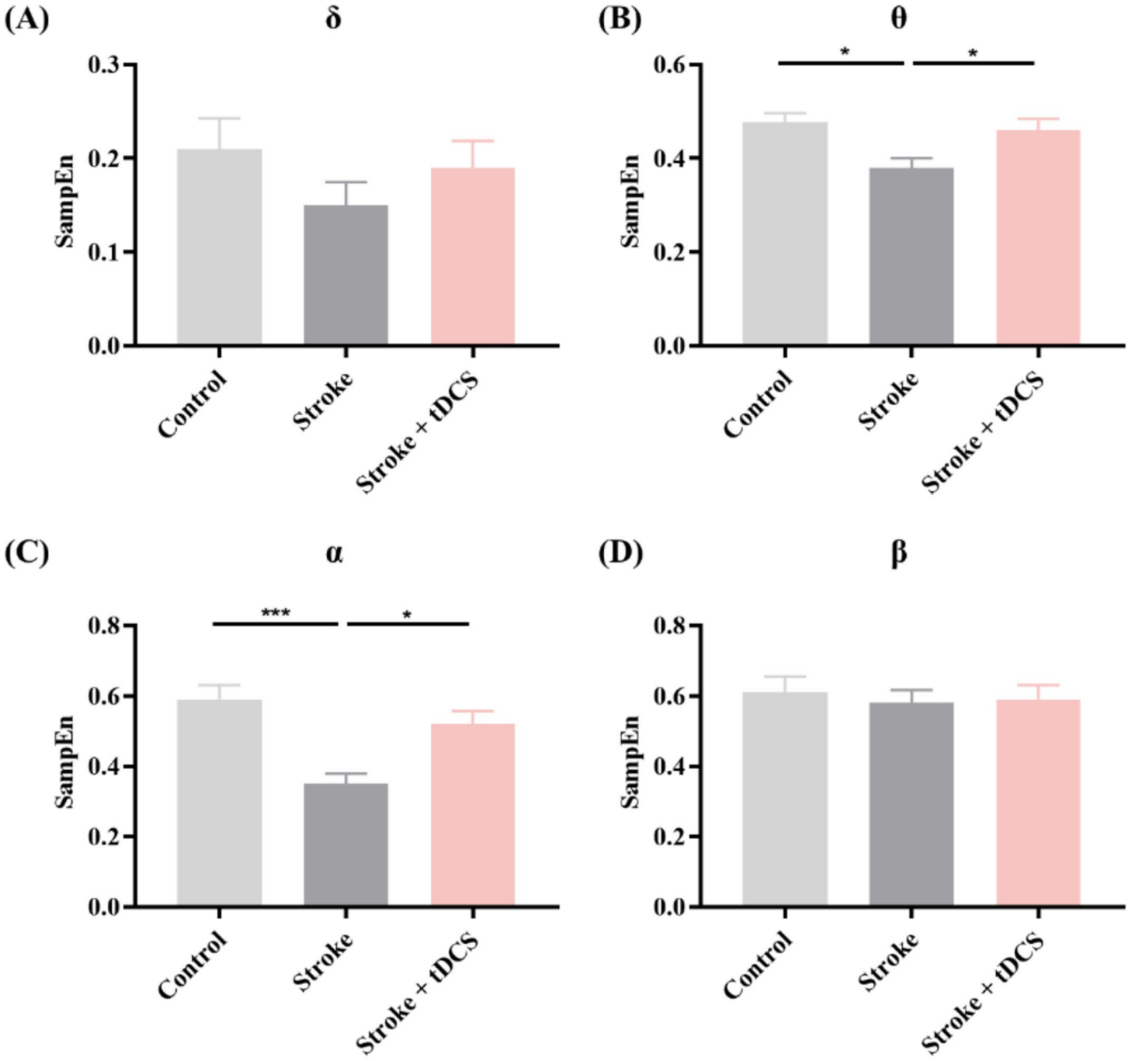
The SampEn in the **(A)** δ, **(B)** θ, **(C)** α, and **(D)** β frequency bands of the clinical groups (mean +SEM, One-way ANOVA, *p < 0.05, ***p < 0.001).

Except for the *β* frequency band, the SampEn of each frequency band in the Stroke group showed a decreasing trend (Figure 6). After the tDCS intervention, the SampEn exhibited an increasing trend, especially in the *θ* and *α* frequency bands (mean ± SEM, One-way ANOVA, *θ*: *p* < 0.05, η^2^ = 0.433; *α*: *p* < 0.05, η^2^ = 0.614, Figure 6).

These results indicated that tDCS effectively reduces the synchronization of neuronal activity in animals and clinical patients with ischemic stroke, aiding their recovery.

## 4 Discussion

This research systematically explored the impacts of tDCS on EEG activity and neural complexity after ischemic stroke by combining animal and clinical experiments. The results demonstrated that the tDCS intervention notably reduced the RP of low-frequency (1–4 Hz) EEG, while increasing the activity of high-frequency (8–30 Hz) bands, accompanied by an increase in the LZC and the SampEn. These findings suggested that tDCS may promote the dynamic reorganization of post-stroke neural networks by regulating the balance of EEG rhythms, providing a potential biomarker for post-stroke neurological recovery.

From the perspective of neural oscillations, the tDCS may inhibit over-synchronized low-frequency oscillations following a stroke by enhancing local excitability (Liu M. et al., 2023). In line with this, the increase in high-frequency beta wave activity may indicate improved efficiency in neural network information processing, which is consistent with clinical observations of motor function recovery in patients (Espenhahn et al., 2020; Schulz et al., 2021; Tang et al., 2020). This bidirectional regulatory effect of the ‘low-frequency inhibition-high-frequency enhancement’ may be the key mechanism by which tDCS to improve post-stroke neural network imbalance.

The synchronous increase in LZC and SampEn further reveals the promoting effect of tDCS on neural information complexity. As nonlinear dynamic indicators, the two reflect the information processing ability of neural networks from the perspectives of algorithmic complexity (LZC) (Fernández et al., 2012) and temporal unpredictability (SampEn) (Bajić and Japundžić-Žigon, 2021), respectively. Additionally, the increase in complexity following the tDCS intervention may be related to the enhancement of neuroplasticity (Longo et al., 2022). This finding theoretically complements the notion that tDCS promotes intercortical functional connectivity (Hordacre et al., 2018; Lefebvre et al., 2017) and together furnishes a firmer theoretical basis for its therapeutic use in stroke.

However, this study still has certain limitations. First, there are differences in brain anatomy and metabolism between animal models and human patients, and the universality of cross-species conclusions needs further verification. Second, the optimization of stimulation parameters, such as current intensity and treatment duration, has not been systematically explored, which may affect the stability of the therapeutic effect. Additionally, the lack of long-term follow-up data limits the evaluation of the persistence of the effect. Future studies should further analyze the mechanisms of tDCS in regulating ischemic stroke by using multimodal brain imaging combined with EEG monitoring. Meanwhile, translate the optimized tDCS stimulation parameters obtained from animal experiments into a plan suitable for human patients through a series of clinical trials. Furthermore, long-term follow-up should be incorporated to comprehensively assess the long-term efficacy and safety of tDCS interventions. This approach would address the current gap in evaluating persistent effects and enhance the clinical translational value of the research.

In summary, this study confirmed through the dual perspectives of frequency domain analysis and nonlinear dynamics that tDCS can regulate the EEG rhythm and enhance neural complexity after stroke. This finding not only provides electrophysiological evidence for the clinical application of tDCS, but also lays a theoretical foundation for the development of individualized neural regulation programs that utilize EEG feedback. Subsequent studies can further explore the synergistic effect of tDCS and other rehabilitation methods to optimize the effect of stroke rehabilitation.

## 5 Conclusion

In summary, tDCS can facilitate the recovery of ischemic stroke by modulating EEG rhythm.

## Data Availability

The original contributions presented in the study are included in the article/supplementary material, further inquiries can be directed to the corresponding author.
